# Cocaine or Methamphetamine Use During Young Adulthood Following Stimulant Use for Attention-Deficit/Hyperactivity Disorder During Adolescence

**DOI:** 10.1001/jamanetworkopen.2023.22650

**Published:** 2023-07-11

**Authors:** Sean Esteban McCabe, John E. Schulenberg, Timothy E. Wilens, Ty S. Schepis, Vita V. McCabe, Phil T. Veliz

**Affiliations:** 1 Center for the Study of Drugs, Alcohol, Smoking and Health, Department of Health Behavior and Biological Sciences, University of Michigan, Ann Arbor; 2 Institute for Research on Women and Gender, University of Michigan, Ann Arbor; 3 Institute for Social Research, University of Michigan, Ann Arbor; 4 Institute for Healthcare Policy and Innovation, University of Michigan, Ann Arbor; 5 Department of Psychology, University of Michigan, Ann Arbor; 6 Department of Psychiatry, Massachusetts General Hospital, Boston; 7 Department of Psychiatry, Harvard Medical School, Harvard University, Boston, Massachusetts; 8 Department of Psychology, Texas State University, San Marcos; 9 Department of Psychiatry, University of Michigan, Ann Arbor

## Abstract

**Importance:**

The prescribing of stimulant medications for attention-deficit/hyperactivity disorder (ADHD) has increased in the US. Prescription stimulants are one of the most commonly misused controlled substances during adolescence. Despite a 10-fold increase in stimulant-related overdose deaths in the past decade, the transitions from prescription stimulants to illicit stimulants (eg, cocaine, methamphetamine) remain relatively unknown in longitudinal population-based studies.

**Objective:**

To determine the longitudinal transitions from adolescents’ prescription stimulant exposure (ie, stimulant therapy for ADHD and prescription stimulant misuse [PSM]) to later cocaine and methamphetamine use during young adulthood.

**Design, Setting, and Participants:**

National longitudinal multicohort panels of US 12th grade public and private school students in the coterminous US were assessed annually (baseline cohort years 2005-2017 [between March and June]) and followed up across 3 waves over a 6-year period to 23 to 24 years of age (follow-up years 2011-2021 [between April and October]).

**Exposure:**

History of self-reported stimulant therapy for ADHD at baseline.

**Main Outcomes and Measures:**

Incidence and prevalence of past-year use of cocaine and methamphetamine during young adulthood (19-24 years of age).

**Results:**

Among 5034 students enrolled at baseline (2589 [52.0%] female), 470 (10.2% [95% CI, 9.4%-11.2%]) reported use of stimulant therapy for ADHD, 671 (14.6% [95% CI, 13.5%-15.6%]) reported PSM only, and 3459 (75.2% [95% CI, 73.9%-76.4%]) reported neither (and served as population controls). In controlled analyses, there were no statistically significant differences between adolescents who reported stimulant therapy for ADHD at baseline compared with population controls in the adjusted odds of transitioning to later cocaine or methamphetamine initiation or use during young adulthood (19-24 years of age). In contrast, PSM during adolescence in those not treated with stimulants for ADHD had significantly higher odds of transitioning to later cocaine or methamphetamine initiation and use during young adulthood compared with population controls (adjusted odds ratio, 2.64 [95% CI, 1.54-4.55]).

**Conclusions and Relevance:**

In this multicohort study, adolescents’ stimulant therapy for ADHD was not associated with increased risk of later cocaine and methamphetamine use during young adulthood. Adolescents’ prescription stimulant misuse offered a signal for subsequent cocaine or methamphetamine use and warrants monitoring and screening.

## Key Points

**Question:**

Is adolescents’ use of stimulant therapy for attention-deficit/hyperactivity disorder (ADHD) associated with cocaine and methamphetamine use as young adults?

**Findings:**

In this cohort study of 5034 respondents, adolescents who used stimulant therapy for ADHD did not differ from their peers in cocaine and methamphetamine use at 19 to 24 years of age. In contrast, adolescents’ prescription stimulant misuse was associated with greater adjusted odds of later cocaine and methamphetamine use compared with population controls.

**Meaning:**

These findings suggest that adolescents’ use of stimulant therapy was not associated with increased risk for illicit stimulant use; however, prescription stimulant misuse was a signal for cocaine or methamphetamine use and warrants monitoring and screening.

## Teaser

This cohort study examines whether prescription stimulant therapy for attention-deficit/hyperactivity disorder (ADHD) and prescription stimulant misuse among adolescents is associated with longitudinal transitions to illicit stimulant use during young adulthood.

## Introduction

The diagnosis of attention-deficit/hyperactivity disorder (ADHD) and stimulant therapy for ADHD have increased substantially over the past 2 decades.^1,2,3^ Although prescription stimulants are effective when used correctly, controversies perplex the clinical and public health fields as prescription stimulants have emerged as a leading controlled medication class misused among US adolescents and young adults.^4,5,6^ Along with the increase in prescription stimulant therapy, there has been a 10-fold increase in overall stimulant-related overdose deaths, calling into question the role of prescription stimulants in the initiation of illicit stimulant use.^7^

Despite high exposure to prescription stimulants during adolescence via stimulant therapy for ADHD and prescription stimulant misuse (PSM), longitudinal transitions from prescription stimulants to illicit stimulants remain relatively unknown in population-based studies. Addressing this gap is particularly important during the developmental period from adolescence to adulthood when individuals become more responsible for their medication management and illicit substance use escalates.^5^ Most individuals who engage in PSM or illicit stimulant use also engage in polysubstance use.^8,9^ To address the knowledge gaps, the main objective of this study is to examine adolescents who are prescribed stimulant therapy for ADHD and those with PSM and their longitudinal transitions to later illicit stimulant use (ie, cocaine or methamphetamine) during young adulthood compared with adolescents who were not prescribed stimulant therapy for ADHD.

## Methods

The Monitoring the Future (MTF) panel study is a multicohort US national longitudinal study of adolescents followed up into adulthood. Procedures and measures are consistent across time; details about methods and procedures are available elsewhere.^5,6^ For the present study, 5034 12th graders (baseline cohort years 2005-2017 [between March and June]) were assessed at baseline and followed up across 3 subsequent survey waves over 6 years to 23 and 24 years of age (follow-up years 2011-2021 [between April and October]; weighted retention rate, 55.3%) (Figure).^5^ These respondents were randomized to 1 of 6 survey forms that included questions about stimulant therapy for ADHD, PSM, cocaine, and methamphetamine. The institutional review board of the University of Michigan deemed this study exempt from approval and the need for informed consent due to the use of deidentified data. This study followed the Strengthening the Reporting of Observational Studies in Epidemiology (STROBE) reporting guidelines for a cohort study.

**Figure.**
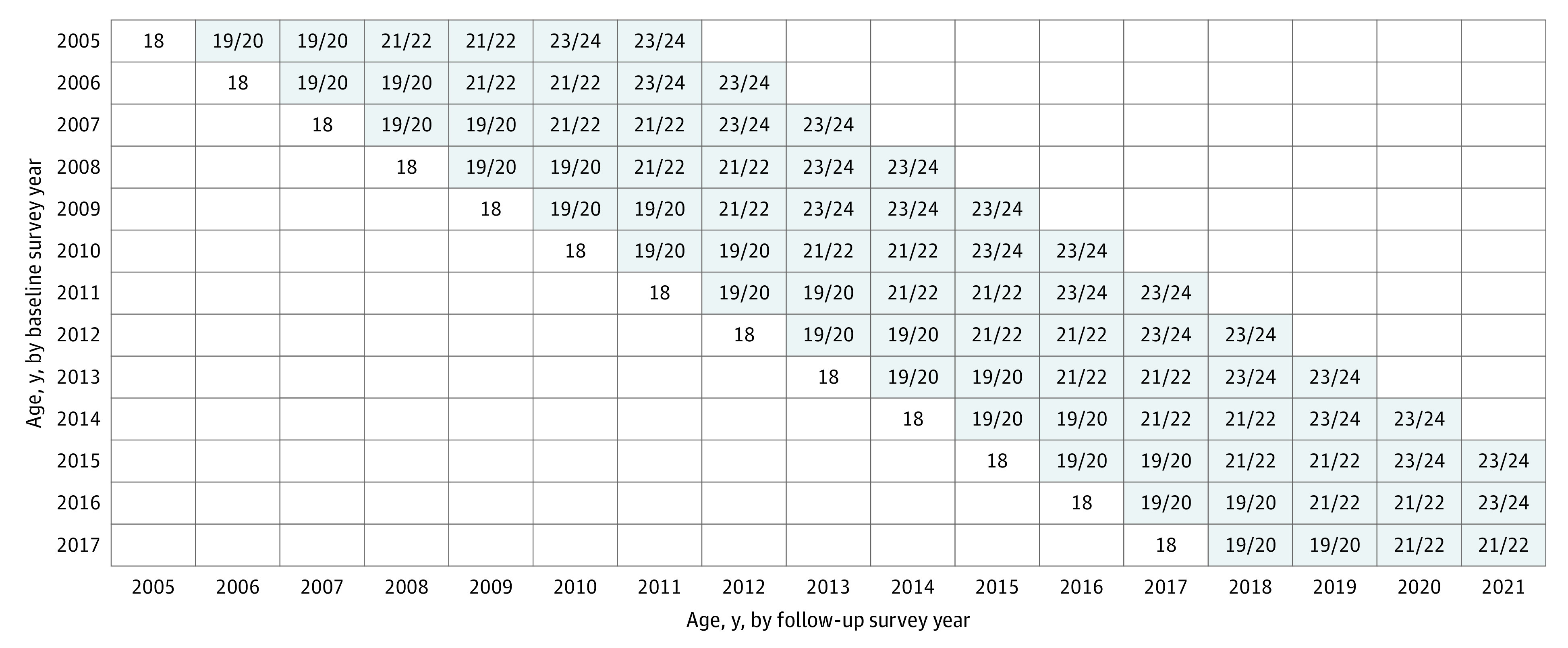
Study Design

### Measures

Stimulant therapy for ADHD was measured at baseline by asking respondents if they had ever taken a prescription stimulant medication for ADHD under a physician’s supervision, such as methylphenidate hydrochloride (Ritalin), combined dextroamphetamine and amphetamine (Adderall), extended-release methylphenidate (Concerta), dextroamphetamine sulfate (Dexedrine), and/or other trade and generic stimulant medications. Respondents were instructed that these medications do not include nonstimulants such as atomoxetine hydrochloride (Strattera), guanfacine hydrochloride (Intuniv), and bupropion hydrochloride (Wellbutrin). The 3 possible response options included no; yes, in the past; and yes, current use.

Prescription stimulant misuse was assessed at baseline and each follow-up survey year by asking respondents on how many occasions (if any) they used prescription stimulants without a physician’s orders in a specific period (lifetime, past year, and past month). Respondents were provided a list of commonly misused prescription stimulants and informed that these are often prescribed for ADHD. The response options for each period ranged from no occasions (option 1) to 40 or more occasions (option 7). Cocaine or methamphetamine use was assessed at baseline and each follow-up survey wave by asking respondents on how many occasions (if any) they used cocaine or methamphetamine (asked separately) in a specific period (lifetime, past year, or past month), using the same response scale as for PSM. Covariates at baseline included self-reported sex, race and ethnicity, grade point average in high school, parental educational level, geographical region, population density, cohort year, binge drinking, cigarette use, marijuana use, prescription opioid misuse, cocaine use, use of nonstimulant therapy for ADHD, and discontinued use of stimulant therapy for ADHD (refers to individuals who reported past but not current use of stimulant therapy for ADHD).

### Statistical Analysis

Unadjusted odds ratios (ORs) and adjusted ORs (AORs) were generated in Stata, version 17.0 (StataCorp LLC)^10^ to examine the associations between the use of stimulant therapy for ADHD and initiation of PSM at 18 years or older and the use of cocaine or methamphetamine at 19 to 24 years of age. Logistic regression models were fitted using the generalized estimating equations methods^11,12^ with an exchangeable correlation structure to assess the association between history of stimulant therapy for ADHD and PSM at baseline (time invariant) and cocaine or methamphetamine initiation or use (time-varying outcomes) at 19 to 24 years of age. Models assessed both the prevalence (using the full panel sample) and incidence (these models excluded respondents with any history of cocaine or methamphetamine use at baseline) of past-year cocaine and methamphetamine use. Both unadjusted and adjusted models were assessed; AORs and 95% CIs were reported in the generalized estimating equations models. All binary logistic regression models were weighted to adjust for differential attrition at follow-ups; item missingness was handled using listwise deletion. The analysis only considers results at 2-sided α ≤ .001 as statistically significant.

## Results

Among 5034 participants at baseline (2589 [52.0% (95% CI, 50.5%-53.3%)] female and 2394 [48.0% (95% CI, 46.6%-49.4%)] male [sex was missing for 51 participants]), an estimated 10.2% (95% CI, 9.4%-11.2%) of adolescents reported lifetime use of stimulant therapy for ADHD (including 294 [6.4% (95% CI, 5.7%-7.1%)] with no PSM history and 176 [3.8% (95% CI, 3.3%-4.4%)] with a PSM history), 671 (14.6% [95% CI, 13.5%-15.6%]) reported PSM only, and 3459 (75.2% [95% CI, 73.9%-76.4%]) did not report stimulant therapy use or PSM and served as population controls. In terms of race and ethnicity, 721 participants (14.3% [95% CI, 13.3%-15.3%]) were Hispanic, 49 (1.0% [95% CI, 0.7%-1.3%]) were non-Hispanic American Indian or Alaska Native, 177 (3.5% [95% CI, 3.0%-4.1%]) were non-Hispanic Asian, 548 (10.9% [95% CI, 10.1%-11.8%]) were non-Hispanic Black, 2747 (54.6% [95% CI, 53.1%-55.9%]) were non-Hispanic White, and 792 (15.7% [95% CI, 14.7%-16.8%]) were other race or ethnicity. Table 1 includes baseline sample demographic, health, and education characteristics.

**Table 1.** Baseline Respondent Characteristics at 18 Years of Age

Characteristic	No. (%) of respondents (N = 5034)^a^
Lifetime prescription stimulant exposure	
No stimulant therapy for ADHD or PSM	3459 (75.2)
Stimulant therapy for ADHD only	294 (6.4)
Stimulant therapy for ADHD and PSM	176 (3.8)
PSM only	671 (14.6)
Lifetime methamphetamine use	
No methamphetamine use (illicit)	4800 (96.4)
Methamphetamine use (illicit)	180 (3.6)
Lifetime cocaine use	
No cocaine use	4489 (90.2)
Cocaine use	487 (9.8)
Sex	
Male	2394 (48.0)
Female	2589 (52.0)
Race and ethnicity	
Hispanic	721 (14.3)
Non-Hispanic American Indian or Alaska Native	49 (1.0)
Non-Hispanic Asian	177 (3.5)
Non-Hispanic Black	548 (10.9)
Non-Hispanic White	2747 (54.6)
Other^b^	792 (15.7)
High school grade point average	
B minus or higher	4032 (82.4)
C plus or lower	859 (17.6)
Parents’ level of education	
Less than a college degree	2319 (48.0)
College degree or higher	2513 (52.0)
Urbanicity	
Large MSA (urban)	1484 (29.5)
Other MSA (suburban)	2463 (48.9)
Non-MSA (rural)	1087 (21.6)
US region	
Northeast	892 (17.7)
Midwest	1221 (24.3)
South	1903 (37.8)
West	1018 (20.2)
Cohort year	
2005-2008	1594 (31.7)
2009-2012	1613 (32.0)
2013-2017	1827 (36.3)

Abbreviations: ADHD, attention-deficit/hyperactivity disorder; MSA, metropolitan statistical area; PSM, prescription stimulant misuse.

^a^ Unweighted sample sizes and percentages are provided. Missing data were 1.2% or lower for all variables such as sex (51 missing cases [1.0%]) except for stimulant therapy and prescription stimulant misuse by 18 years of age (8.6%), grade point average during high school (2.8%), and parents’ level of education (4.9%).

^b^ Includes more than 1 race or ethnicity.

As shown in Table 2, there were no statistically significant differences (in unadjusted and adjusted models) between adolescents who reported use of stimulant therapy for ADHD and adolescents who never used stimulant therapy in the odds of cocaine or methamphetamine use in the prevalence or incidence at 19 to 24 years of age. eTable 1 in Supplement 1 includes the full set of results for each of the covariates that were included in the models. While there were no associations between the baseline sociodemographic characteristics and the outcomes, there were a number of associations between baseline substance-related covariates (eg, marijuana use) and later cocaine and methamphetamine use during young adulthood.

**Table 2.** Adolescents’ Stimulant Therapy for ADHD at 18 Years of Age and Later Cocaine or Methamphetamine Use and Incidence at 19 to 24 Years of Age^a^

Model	Never used ADHD stimulant therapy	Stimulant therapy for ADHD	Time^b^
**Any stimulant therapy for ADHD at 18 years of age**			
Unadjusted model prevalence, weighted % (OR [95% CI])			
Past-year cocaine use (n = 2527)	6.29 (1 [Reference])	7.96 (1.05 [0.65-1.84])	NA (1.17 [1.01-1.37])
Past-year methamphetamine use (n = 2526)	0.90 (1 [Reference])	2.00 (1.38 [0.61-3.13])	NA (1.24 [0.86-1.80])
Past-year cocaine or methamphetamine use (n = 2531)	6.40 (1 [Reference])	8.43 (1.08 [0.63-1.87])	NA (1.18 [1.01-1.38])
Adjusted model prevalence, AOR (95% CI)^c^			
Past-year cocaine use (n = 2198)	1 [Reference]	0.76 (0.38-1.52)	1.16 (0.97-1.39)
Past-year methamphetamine use (n = 2196)	1 [Reference]	1.00 (0.35-2.85)	1.15 (0.78-1.71)
Past-year cocaine or methamphetamine use (n = 2201)	1 [Reference]	0.79 (0.40-1.54)	1.15 (0.96-1.39)
**Stimulant therapy use for ADHD, excluding those who reported lifetime cocaine or methamphetamine use at 18 years of age**			
Unadjusted model incidence, weighted % (OR [95% CI])			
Cocaine use (n = 2295)^d^	4.85 (1 [Reference])	4.78 (1.06 [0.49-2.31])	NA (1.32 [1.09-1.60])
Methamphetamine use (n = 2294)^e^	0.55 (1 [Reference])	0.56 (0.60 [0.14-2.64])	NA (1.63 [0.94-2.83])
Cocaine or methamphetamine use (n = 2298)^f^	4.84 (1 [Reference])	5.07 (1.09 [0.51-2.32])	NA (1.33 [1.10-1.61])
Adjusted model incidence, AOR (95% CI)			
Cocaine use (n = 2006)^d^	1 [Reference]	0.56 (0.20-1.56)	1.34 (1.07-1.68)
Methamphetamine use (n = 2004)^e^	1 [Reference]	0.23 (0.04-1.48)	1.75 (0.90-3.38)
Cocaine or methamphetamine use (n = 2004)^f^	1 [Reference]	0.59 (0.22-1.59)	1.34 (1.07-1.68)

Abbreviations: ADHD, attention-deficit/hyperactivity disorder; AOR, adjusted odds ratio; NA, not applicable; OR, odds ratio.

^a^ Unweighted sample sizes are provided. All estimates provided use weights to adjust for attrition.

^b^ Indicates 19 to 20 years of age (0) to 23 to 24 years of age (2).

^c^ Included the following time-invariant covariates: sex, race and ethnicity, parents’ level of education, urbanicity, US region, cohort year, grade point average during high school, past-30-day cigarette use (18 years of age), past-2-week binge drinking (18 years of age), past-year marijuana use (18 years of age), past-year prescription opioid misuse (18 years of age), past-year prescription stimulant misuse (18 years of age), lifetime cocaine use (18 years of age), lifetime methamphetamine use (18 years of age), lifetime use of nonstimulant therapy for ADHD (18 years of age), and discontinued use of stimulant therapy for ADHD (18 years of age). eTable 1 in Supplement 1 provides results associated with all covariates.

^d^ Excluded individuals who reported lifetime cocaine use at 18 years of age; the same control variables for models for adjusted prevalence of past-year cocaine and/or methamphetamine use were included for adjusted incidence of cocaine use (excluding lifetime cocaine use at 18 years of age).

^e^ Excluded individuals who reported lifetime methamphetamine use at 18 years of age; the same control for adjusted prevalence of past-year cocaine and/or methamphetamine use was included for adjusted incidence of methamphetamine use (excluding lifetime methamphetamine use at 18 years of age).

^f^ Excluded individuals who reported cocaine or methamphetamine use at 18 years of age; the same control variables for adjusted prevalence of past-year cocaine and/or methamphetamine use were included for adjusted incidence of cocaine or methamphetamine use (excluding lifetime cocaine use and methamphetamine use at 18 years of age).

As shown in Table 3, adolescents with PSM had the highest unadjusted and adjusted odds of later cocaine and methamphetamine use at 19 to 24 years of age in terms of prevalence and incidence vs population controls. Adolescents who reported PSM with no history of stimulant therapy for ADHD at baseline had more than 2.5 times greater adjusted odds than population controls of cocaine or methamphetamine initiation (AOR, 2.64 [95% CI, 1.54-4.55]) at 19 to 24 years of age. eTable 2 in Supplement 1 provides the full set of results for each of the covariates that were included in the models. Although there were no associations between the baseline sociodemographic characteristics and the outcomes, there were a number of baseline substance-related covariates (eg, marijuana use) that were associated with later cocaine and methamphetamine use during young adulthood.

**Table 3.** Adolescents’ Prescription Stimulant Exposure at 18 Years of Age and Later Cocaine or Methamphetamine Use and Incidence at 19 to 24 Years of Age^a^

Model	No prescription stimulant use or misuse	Use of stimulant therapy for ADHD only	Use of stimulant therapy for ADHD and misuse	Prescription stimulant misuse only	Time^b^
**Prescription stimulant exposure at 18 years of age**					
Unadjusted model prevalence, weighted % (OR [95% CI])					
Past-year cocaine use (n = 2527)	4.20 (1 [Reference])	5.62 (1.67 [0.79-3.54])	15.00 (2.44 [1.19-4.98])	23.98 (5.44 [3.49-8.47])	NA (1.19 [1.01-1.41])
Past-year methamphetamine use (n = 2526)	0.43 (1 [Reference])	0.66 (1.67 [0.34-8.29])	6.25 (7.49 [2.37-23.72])	4.71 (7.26 [2.61-20.11])	NA (1.35 [0.93-1.97])
Past-year cocaine or methamphetamine use (n = 2531)	4.19 (1 [Reference])	6.25 (1.75 [0.86-3.59])	16.00 (2.46 [1.21-5.00])	24.83 (5.44 [3.49-8.46])	NA (1.19 [1.01-1.41])
Adjusted model prevalence, AOR (95% CI)^c^					
Past-year cocaine use (n = 2198)	1 [Reference]	1.41 (0.56-3.57)	0.87 (0.39-1.95)	1.90 (1.17-3.09)	1.16 (0.97-1.40)
Past-year methamphetamine use (n = 2196)	1 [Reference]	1.54 (0.24-9.72)	2.82 (0.62-12.70)	3.32 (0.97-10.84)	1.16 (0.78-1.72)
Past-year cocaine or methamphetamine use (n = 2201)	1 [Reference]	1.51 (0.63-3.67)	0.86 (0.38-1.91)	1.93 (1.19-3.13)	1.16 (0.96-1.39)
**Prescription stimulant exposure at 18 years of age, excluding those who reported lifetime cocaine or methamphetamine use at baseline**					
Unadjusted model incidence, weighted % (OR [95% CI])					
Cocaine use (n = 2295)^d^	3.53 (1 [Reference])	4.45 (1.55 [0.61-3.95])	6.34 (1.86 [0.64-5.44])	19.81 (7.75 [4.89-12.23])	NA (1.35 [1.10-1.66])
Methamphetamine use (n = 2294)^e^	0.36 (1 [Reference])	0.34 (0.67 [0.08-5.41])	1.58 (3.81 [0.49-29.84])	3.00 (10.51 [3.59-31.10])	NA (1.70 [0.98-2.99])
Cocaine or methamphetamine use (n = 2282)^f^	3.53 (1 [Reference])	4.45 (1.54 [0.61-3.93])	7.93 (2.13 [0.80-5.70])	19.81 (7.79 [4.92-12.30])	NA (1.36 [1.11-1.67])
Adjusted model incidence, AOR (95% CI)					
Cocaine use (n = 2006)^d^	1 [Reference]	1.06 (0.33-3.46)	0.53 (0.16-1.75)	2.59 (1.51-4.46)	1.35 (1.07-1.71)
Methamphetamine use (n = 2004)^e^	1 [Reference]	0.27 (0.02-3.11)	0.96 (0.06-14.84)	4.66 (0.91-23.71)	1.75 (0.89-3.43)
Cocaine or methamphetamine use (n = 2008)^f^	1 [Reference]	1.06 (0.33-3.46)	0.65 (0.22-1.93)	2.64 (1.54-4.55)	1.35 (1.07-1.70)

Abbreviations: ADHD, attention-deficit/hyperactivity disorder; AOR, adjusted odds ratio; NA, not applicable; OR, odds ratio.

^a^ Unweighted sample sizes are provided. All estimates provided use weights to adjust for attrition.

^b^ Indicates 19 to 20 years of age (0) to 23 to 24 years of age (2).

^c^ Included the following time-invariant covariates: sex, race and ethnicity, parents’ level of education, urbanicity, US region, cohort year, grade point average during high school, past-30-day cigarette use (18 years of age), past-2-week binge drinking (18 years of age), past-year marijuana use (18 years of age), past-year prescription opioid misuse (18 years of age), lifetime cocaine use (18 years of age), lifetime methamphetamine use (18 years of age), lifetime use of nonstimulant therapy for ADHD (18 years of age), and discontinued use of stimulant therapy for ADHD (18 years of age). Please refer to eTable 2 in Supplement 1 for results associated with all covariates.

^d^ Excluded individuals who reported lifetime cocaine use at 18 years of age; the same control variables were adjusted for past-year prevalence of cocaine and/or methamphetamine use were included adjusted incidence of cocaine use (excluding lifetime cocaine use at 18 years of age).

^e^ Excluded individuals who reported lifetime methamphetamine use at 18 years of age; the same control variables were adjusted for past-year prevalence of cocaine and/or methamphetamine use were included for adjusted incidence of methamphetamine use (excluding lifetime methamphetamine use at 18 years of age).

^f^ Excluded individuals who reported cocaine or methamphetamine use at 18 years of age; the same control variables were adjusted for past-year prevalence of cocaine and/or methamphetamine use were included for adjusted incidence of cocaine or methamphetamine use (excluding lifetime cocaine and methamphetamine use at 18 years of age).

Sensitivity analyses were performed and showed that current use of stimulant therapy for ADHD was not associated with later methamphetamine or cocaine use or initiation in any model compared with population controls who never used or misused prescription stimulants (eTable 3 in Supplement 1). Sensitivity analyses were conducted to examine whether baseline PSM frequency was associated with later incidence or prevalence of cocaine or methamphetamine use at 19 to 24 years of age. For example, among respondents who engaged in baseline PSM with no lifetime history of medical stimulant use, cocaine or methamphetamine use was more prevalent as a function of lifetime PSM frequency ranging from 17.7% (95% CI, 11.1%-26.8%) among those who reported PSM 1 or 2 times, 28.4% (95% CI, 19.2%-39.9%) for PSM 3 to 9 times, and 34.1% (95% CI, 24.7%-44.8%) for PSM 10 or more times. In controlled sensitivity analyses, adolescents who reported past-year PSM 10 or more times with no history of stimulant therapy use for ADHD at baseline had more than 4 times greater adjusted odds of indicating cocaine or methamphetamine use compared with population controls (AOR, 4.27 [95% CI, 1.78-10.25]) at 19 to 24 years of age. Adolescents who reported past-year PSM 1 or 2 times with no history of stimulant therapy for ADHD at baseline had more than 2.5 times greater adjusted odds of indicating cocaine or methamphetamine use when compared with population controls (AOR, 2.60 [95% CI, 1.27-5.32]) at 19 to 24 years of age. We found similar results for incidence of cocaine or methamphetamine use. In controlled analyses, adolescents who reported past-year PSM 1 or 2 times with no history of stimulant therapy for ADHD at baseline had 3 times greater adjusted odds of indicating incident cocaine or methamphetamine use when compared with population controls (AOR, 3.00 [95% CI, 1.26-7.71]) at 19 to 24 years of age.

## Discussion

Based on the increases in prescription stimulant therapy for ADHD and psychostimulant-involved overdoses, there is a growing need for public health practitioners and researchers to understand whether exposure to stimulant therapy for ADHD is associated with later illicit stimulant use. This study found that adolescents’ stimulant therapy for ADHD was not associated with cocaine or methamphetamine use during young adulthood. While ADHD is a risk factor for illicit stimulant use and substance use disorders,^13^ the present study offers promising results for adolescents who benefit from stimulant therapy for ADHD. The present cohort study found that adolescents’ stimulant therapy for ADHD was associated with prevalence and incidence of illicit stimulant use that was similar to their peers who never used stimulant therapy for ADHD.

Prescription stimulant misuse during adolescence in those not treated with stimulants for ADHD was associated with more than 2 times greater odds of incident cocaine and methamphetamine use during young adulthood (eg, 20% incident cocaine use). Given the high prevalence of PSM in adolescence (>18%), this information supplements the current black box labeling on prescription stimulants regarding addiction liability and warrants additional attention regarding risk reduction strategies.^14^ The present study found that cocaine or methamphetamine use was reported by about 1 in 3 (34.1%) individuals during young adulthood (19-24 years of age) among those adolescents who reported PSM 10 or more times. These findings support monitoring adolescents for PSM and inclusion of PSM in brief screening instruments (eg, SBIRT [Screening, Brief Intervention, and Referral to Treatment] or the TAPS [Tobacco, Alcohol, Prescription Medication, and Other Substance Use] Tool) to determine whether education, brief intervention, or more comprehensive assessment and intensive management is required following screening, despite the US Preventive Services Task Force not currently recommending screening for unhealthy drug use during adolescence.^15^ The inclusion of PSM in screening instruments seems especially relevant during the transition from adolescence into young adulthood, considering the present study found in controlled analyses that adolescents who reported infrequent past-year PSM with no history of stimulant therapy for ADHD at baseline had 3 times greater adjusted odds of indicating incident cocaine or methamphetamine use when compared with population controls at 19 to 24 years of age. The present study also found associations between baseline marijuana use and later cocaine and methamphetamine use that deserve more attention in monitoring and screening efforts.

### Strengths and Limitations

The main strength of the present study is the nationally representative sample of 12th grade students attending US public and private schools with large enough samples to examine exposure to stimulant therapy for ADHD and PSM over time. Limitations of the MTF study include school-based samples that exclude youths who drop out of school or are home-schooled, potential attrition bias, self-report medication therapy, potential unassessed confounders, and treatment differences. Although self-report data in the MTF study generally have been found to be reliable and valid, studies suggest that misclassification and underreporting of substance use do occur.^5,6^ The MTF study attempted to minimize such bias by using conditions that past research^5,6^ has been shown to improve the validity and reliability of substance use data collected via self-report surveys, such as explaining the relevance of the study and informing potential respondents about voluntary participation and anonymity. In the MTF study, no adjustments are made to correct for any underreporting; thus, results from the present study may be conservative and underreport the actual prevalence of sensitive behaviors. The MTF study does not assess some factors that could be associated with stimulant misuse (eg, ADHD severity, medication dose and adherence, nonpharmacological therapy, deviant peers with access to drugs). The MTF study also did not separate amphetamine from methylphenidate treatments, which is important given potentially different misuse liabilities.

## Conclusions

In this multicohort study of adolescents exposed to prescription stimulants, adolescents who used stimulant therapy for ADHD did not differ from population controls in initiation of illicit stimulant (cocaine or methamphetamine) use, which suggested a potential protective effect, given evidence of elevated illicit stimulant use among those with ADHD. In contrast, monitoring adolescents for PSM is warranted because this behavior offered a strong signal for transitioning to later cocaine or methamphetamine initiation and use during young adulthood.

## Notes

Supplement 1. **eTable 1.** Adolescents’ Stimulant Therapy for ADHD at 18 Years of Age and Later Cocaine or Methamphetamine Use at 19 to 24 Years of Age **eTable 2.** Adolescents’ Prescription Stimulant Exposure at 18 Years of Age and Later Cocaine or Methamphetamine Use and Incidence at 19 to 24 Years of Age **eTable 3. **Adolescents’ Current and Past Stimulant Therapy for ADHD and Prescription Stimulant Misuse at 18 Years of Age and Later Cocaine and Methamphetamine Use at 19 to 24 Years of Age

Supplement 2.
Data Sharing Statement
