# Supplementary material for: Cocaine or Methamphetamine Use During Young Adulthood Following Stimulant Use for Attention-Deficit/Hyperactivity Disorder During Adolescence
Source: JAMA Netw Open. 2023 Jul 11;6(7):e2322650. doi: 10.1001/jamanetworkopen.2023.22650 (PMC10336617; doi:10.1001/jamanetworkopen.2023.22650)
Supplement: Supplement 1. — eTable 1. Adolescents’ Stimulant Therapy for ADHD at 18 Years of Age and Later Cocaine or Methamphetamine Use at 19 to 24 Years of Age eTable 2. Adolescents’ Prescription Stimulant Exposure at 18 Years of Age and Later Cocaine or Methamphetamine Use and Incidence at 19 to 24 Years of Age eTable 3. Adolescents’ Current and Past Stimulant Therapy for ADHD and Prescription Stimulant Misuse at 18 Years of Age and Later Cocaine and Methamphetamine Use at 19 to 24 Years of Age [file jamanetwopen-e2322650-s001.pdf]

Supplementary Online Content

McCabe SE, Schulenberg JE, Wilens TE, Schepis TS, McCabe VV, Veliz PT. Cocaine or methamphetamine use during young adulthood following stimulant use for attention-deficit/hyperactivity disorder during adolescence. *JAMA Netw Open*. 2023;6(7):e2322650. doi:10.1001/jamanetworkopen.2023.22650

eTable 1. Adolescents' Stimulant Therapy for ADHD at 18 Years of Age and Later Cocaine or Methamphetamine Use at 19 to 24 Years of Age
eTable 2. Adolescents' Prescription Stimulant Exposure at 18 Years of Age and Later Cocaine or Methamphetamine Use and Incidence at 19 to 24 Years of Age
eTable 3. Adolescents' Current and Past Stimulant Therapy for ADHD and Prescription Stimulant Misuse at 18 Years of Age and Later Cocaine and Methamphetamine Use at 19 to 24 Years of Age

This supplementary material has been provided by the authors to give readers additional information about their work.

© 2023 McCabe SE et al. *JAMA Network Open*.

eTable 1. Adolescents' Stimulant Therapy for ADHD at 18 Years of Age and Later Cocaine or Methamphetamine Use at 19 to 24 Years of Age

	**Adjusted models, ages 19–24 (Prevalence)**		
	**Past-year cocaine use**	**Past-year methamphetamine use**	**Past-year cocaine or methamphetamine use**
	**Model 4**	**Model 5**	**Model 6**
	**n = 2198**	**n = 2196**	**n = 2201**
	**AOR (95% CI)**[a]	**AOR (95% CI)**[a]	**AOR (95% CI)**[a]
**Stimulant therapy for ADHD at age 18**			
Never used stimulant therapy for ADHD	Reference	Reference	Reference
Stimulant therapy for ADHD	0.76 (0.38, 1.52)	1.00 (0.35, 2.85)	0.79 (0.40, 1.54)
**Time**			
Age (0 = 19/20 to 2 = 23/24)	1.16 (0.97, 1.39)	1.15 (0.78, 1.71)	1.15 (0.96, 1.39)
**Sex**			
Male	Reference	Reference	Reference
Female	0.88 (0.60, 1.31)	1.22 (0.61, 2.44)	0.87 (0.59, 1.28)
**Race**			
White	Reference	Reference	Reference
Non-White	1.17 (0.72, 1.93)	1.43 (0.54, 3.83)	1.17 (0.72, 1.91)
**Parents' level of education**			
Less than a college degree	Reference	Reference	Reference
College degree or higher	1.27 (0.86, 1.88)	1.33 (0.58, 3.08)	1.28 (0.86, 1.90)
**Urbanicity**			
Large MSA (urban)	Reference	Reference	Reference
Other MSA (suburban)	0.89 (0.58, 1.35)	1.80 (0.58, 5.62)	0.89 (0.58, 1.36)
Non-MSA (rural)	0.68 (0.40, 1.15)	2.27 (0.53, 9.64)	0.73 (0.43, 1.24)
**US region**			
Northeast	Reference	Reference	Reference
Midwest	1.07 (0.61, 1.88)	0.56 (0.17, 1.94)	1.07 (0.62, 1.88)
South	0.61 (0.33, 1.10)	0.49 (0.14, 1.69)	0.63 (0.33, 1.15)
West	1.34 (0.78, 2.32)	0.57 (0.21, 1.59)	1.36 (0.79, 2.35)
**Cohort year**			
2005–2008	Reference	Reference	Reference
2009–2012	0.95 (0.61, 1.48)	0.86 (0.33, 2.25)	0.99 (0.63, 1.56)
2013–2017	1.09 (0.66, 1.81)	0.61 (0.19, 1.94)	1.12 (0.67, 1.85)
**Grade point average (GPA) during high school**			
B- or higher	Reference	Reference	Reference
C+ or lower	0.66 (0.38, 1.14)	0.57 (0.19, 1.78)	0.68 (0.39, 1.16)
**Substance Use at Age 18**			
Past-30-day cigarette use	1.86 (1.17, 2.95)	2.18 (0.73, 6.50)	1.87 (1.18, 2.96)
Past-two-week binge drinking	2.42 (1.46, 4.03)	1.14 (0.37, 3.48)	2.45 (1.48, 4.04)

© 2023 McCabe SE et al. *JAMA Network Open*.

Past-year marijuana use	4.22 (2.40, 7.44)	9.28 (1.72, 49.81)	4.30 (2.45, 7.54)
Past-year prescription opioid misuse	1.56 (0.91, 2.70)	1.47 (0.48, 4.55)	1.57 (0.92, 2.69)
Past-year prescription stimulant misuse	2.28 (1.35, 3.84)	2.75 (0.79, 9.55)	2.16 (1.28, 3.63)
Lifetime cocaine use	2.23 (1.31, 3.81)	2.76 (1.22, 6.24)	2.46 (1.44, 4.20)
Lifetime methamphetamine use	0.42 (0.19, 0.90)	1.39 (0.47, 4.11)	.457 (0.22, 0.96)
**History of Non-Stimulant Therapy for ADHD at Age 18**			
Did not use non-stimulant therapy for ADHD	Reference	Reference	Reference
Used non-stimulant therapy for ADHD	1.39 (0.66, 2.95)	1.95 (0.52, 7.39)	1.32 (0.62, 2.83)
**Discontinued Stimulant Therapy for ADHD by Age 18**			
Did not use/did not discontinue use	Reference	Reference	Reference
Discontinued use	1.49 (0.49, 4.49)	0.38 (0.08, 1.74)	1.63 (0.55, 4.82)

Abbreviations: ADHD, attention-deficit/hyperactivity disorder; meth, methamphetamine; OR, odds ratio; CI, confidence interval; AOR, adjusted odds ratio; %, weighted prevalence/incidence.
Notes: Unweighted sample sizes are provided. All estimates provided use weights to adjust for attrition.
[a]Adjusted models 4, 5 and 6 also included the following time-invariant variables: sex, race/ethnicity, parents' level of education, urbanicity, US region, cohort year, grade point average (GPA) during high school, past-30-day cigarette use (age 18), past-two-week binge drinking (age 18), past-year marijuana use (age 18), past-year prescription opioid misuse (age 18), past-year prescription stimulant misuse (age 18), lifetime cocaine use (age 18), lifetime methamphetamine use (age 18), lifetime non-stimulant therapy for ADHD (age 18), and discontinued stimulant therapy for ADHD (age 18). Results for the covariates are similar with respect to models that assessed incidence.

© 2023 McCabe SE et al. *JAMA Network Open*.

eTable 2. Adolescents' Prescription Stimulant Exposure at 18 Years of Age and Later Cocaine or Methamphetamine Use and Incidence at 19 to 24 Years of Age

	**Adjusted models, ages 19–24 (Prevalence)**		
	**Past-year cocaine use**	**Past-year methamphetamine use**	**Past-year cocaine or methamphetamine use**
	**Model 16**	**Model 17**	**Model 18**
	**n = 2198**	**n = 2196**	**n = 2201**
	**AOR (95% CI)**[a]	**AOR (95% CI)**[a]	**AOR (95% CI)**[a]
**Stimulant therapy for ADHD at age 18**			
No prescription stimulant use or misuse	Reference	Reference	Reference
Stimulant therapy for ADHD only	1.41 (0.56, 3.57)	1.54 (0.24, 9.72)	1.51 (0.63, 3.67)
Stimulant therapy for ADHD and misuse	0.87 (0.39, 1.95)	2.82 (0.62, 12.70)	0.86 (0.38, 1.91)
Prescription stimulant misuse only	1.90 (1.17, 3.09)	3.32 (0.97, 10.84)	1.93 (1.19, 3.13)
**Time**			
Age (0 = 19/20 to 2 = 23/24)	1.16 (0.97, 1.39)	1.15 (0.78, 1.71)	1.15 (0.96, 1.39)
**Sex**			
Male	Reference	Reference	Reference
Female	0.91 (0.62, 1.32)	1.23 (0.60, 2.55)	0.88 (0.61, 1.28)
**Race**			
White	Reference	Reference	Reference
Non-White	1.16 (0.72, 1.88)	1.41 (0.53, 3.79)	1.17 (0.73, 1.89)
**Parents' level of education**			
Less than a college degree	Reference	Reference	Reference
College degree or higher	1.26 (0.85, 1.86)	1.35 (0.57, 3.17)	1.27 (0.86, 1.87)
**Urbanicity**			
Large MSA (urban)	Reference	Reference	Reference
Other MSA (suburban)	0.86 (0.56, 1.33)	1.77 (0.57, 5.53)	0.88 (0.57, 1.35)
Non-MSA (rural)	0.63 (0.37, 1.07)	2.26 (0.53, 9.74)	0.68 (0.40, 1.16)
**US region**			
Northeast	Reference	Reference	Reference
Midwest	1.05 (0.59, 1.85)	0.54 (0.15, 1.95)	1.05 (0.60, 1.85)
South	0.62 (0.34, 1.14)	0.52 (0.15, 1.80)	0.64 (0.35, 1.18)
West	1.35 (0.78, 2.36)	0.56 (0.20, 1.59)	1.37 (0.79, 2.38)
**Cohort year**			
2005–2008	Reference	Reference	Reference
2009–2012	0.94 (0.60, 1.48)	0.84 (0.32, 2.24)	0.99 (0.63, 1.56)
2013–2017	1.10 (0.68, 1.78)	0.57 (0.17, 1.87)	1.12 (0.69, 1.81)
**Grade point average (GPA) during high school**			
B- or higher	Reference	Reference	Reference
C+ or lower	0.68 (0.41, 1.13)	0.56 (0.17, 1.81)	0.69 (0.42, 1.16)

© 2023 McCabe SE et al. *JAMA Network Open*.

**Substance Use at Age 18**			
Past-30-day cigarette use	1.85 (1.16, 2.94)	1.88 (0.63, 5.58)	1.84 (1.17, 2.92)
Past-two-week binge drinking	2.55 (1.56, 4.16)	1.18 (0.40, 3.49)	2.56 (1.57, 4.17)
Past-year marijuana use	4.10 (2.34, 7.21)	8.41 (1.57, 45.12)	4.16 (2.38, 7.28)
Past-year prescription opioid misuse	1.77 (1.10, 2.85)	1.64 (0.59, 4.52)	1.75 (1.09, 2.82)
Lifetime cocaine use	2.12 (1.24, 3.64)	2.48 (1.10, 5.61)	2.33 (1.35, 4.02)
Lifetime methamphetamine use	0.52 (0.25, 1.09)	1.51 (0.52, 4.45)	0.55 (0.27, 1.14)
**History of Non-Stimulant Therapy for ADHD at Age 18**			
Did not use non-stimulant therapy for ADHD	Reference	Reference	Reference
Used non-stimulant therapy for ADHD	1.31 (0.63, 2.72)	1.96 (0.49, 7.78)	1.25 (0.60, 2.62)
**Discontinued Stimulant Therapy for ADHD by Age 18**			
Did not use/did not discontinue use	Reference	Reference	Reference
Discontinued use	1.52 (0.51, 4.53)	0.35 (0.07, 1.68)	1.66 (0.58, 4.82)

Abbreviations: ADHD, attention-deficit/hyperactivity disorder; OR, odds ratio; CI, confidence interval; AOR, adjusted odds ratio.
Notes: Unweighted sample sizes are provided. All estimates provided use weights to adjust for attrition.
[a]Adjusted models 16-18 included the following time-invariant variables: sex, race/ethnicity, parents' level of education, urbanicity, US region, cohort year, grade point average (GPA) during high school, past-30-day cigarette use (age 18), past-two-week binge drinking (age 18), past-year marijuana use (age 18), past-year prescription opioid misuse (age 18), lifetime cocaine use (age 18), lifetime methamphetamine use (age 18), lifetime non-stimulant therapy for ADHD (age 18), and discontinued stimulant therapy for ADHD (age 18). Results for the covariates are similar with respect to models that assessed incidence.

© 2023 McCabe SE et al. *JAMA Network Open*.

eTable 3. Adolescents' Current and Past Stimulant Therapy for ADHD and Prescription Stimulant Misuse at 18 Years of Age and Later Cocaine and Methamphetamine Use at 19 to 24 Years of Age

	**Unadjusted models**	**Adjusted models**
	**Ages 19–24 past-year cocaine or methamphetamine use (prevalence)**	**Ages 19–24 past-year cocaine or methamphetamine use (prevalence)**
	**n = 2508 OR (95% CI)**	**n = 2201 AOR (95% CI)[a]**
**Stimulant therapy and misuse (full sample, age 18)**		
Never used or misused prescription stimulants	Reference	Reference
Current stimulant therapy for ADHD only	0.50 (0.16, 1.58)	0.49 (0.14, 1.72)
Past stimulant therapy for ADHD only	2.58 (1.17, 5.71)	2.43 (0.83, 7.12)
Current stimulant therapy for ADHD and misuse	4.52 (2.09, 9.78)	1.30 (0.47, 3.65)
Past stimulant therapy for ADHD and misuse	1.75 (0.68, 4.58)	0.71 (0.26, 1.95)
Prescription stimulant misuse only	5.40 (3.47, 8.41)	1.92 (1.18, 3.11)
**Time**		
Age (0 = 19/20 to 2 = 23/24)	1.19 (1.01, 1.40)	1.16 (0.96, 1.39)
	**Unadjusted models**	**Adjusted models**
	**Ages 19–24 cocaine or methamphetamine use (incidence)[a]**	**Ages 19–24 cocaine or methamphetamine use (incidence)[a]**
	**n = 2282 OR (95% CI)**	**n = 2008 AOR (95% CI)**
**Stimulant therapy and misuse at age 18 (models excluded youth who reported lifetime cocaine or methamphetamine use at age 18)**		
Never used or misused prescription stimulants	Reference	Reference
Current stimulant therapy for ADHD only	0.23 (0.03, 1.69)	0.26 (0.04, 1.89)
Past stimulant therapy for ADHD only	2.39 (0.90, 6.34)	1.72 (0.42, 7.07)
Current stimulant therapy for ADHD and misuse	2.05 (0.49, 8.67)	0.54 (0.10, 2.84)
Past stimulant therapy for ADHD and misuse	2.18 (0.60, 7.96)	0.78 (0.22, 2.77)
Prescription stimulant misuse only	7.80 (4.92, 12.30)	2.63 (1.53, 4.52)
**Time**		
Age (0 = 19/20 to 2 = 23/24)	1.36 (1.11, 1.66)	1.35 (1.07, 1.71)

Abbreviations: ADHD, attention-deficit/hyperactivity disorder; OR, odds ratio; CI, confidence interval; AOR, adjusted odds ratio.
Notes: Unweighted sample sizes are provided. All estimates provided use weights to adjust for attrition at first follow-up (ages 19/20). Adjusted models control for the following time-invariant variables: sex, race/ethnicity, parents' level of education, urbanicity, US region, cohort year, grade point average (GPA) during high school, truancy, past-30-day cigarette use (age 18), past-two-week binge drinking (age 18), past-year marijuana use (age 18), past-year prescription opioid misuse (age 18), and non-stimulant therapy for ADHD (age 18).
[a]Models exclude individuals who reported lifetime cocaine or methamphetamine use at age 18.

© 2023 McCabe SE et al. *JAMA Network Open*.
