# Supplementary material for: Cocaine or Methamphetamine Use During Young Adulthood Following Stimulant Use for Attention-Deficit/Hyperactivity Disorder During Adolescence
Source: JAMA Netw Open. 2023 Jul 11;6(7):e2322650. doi: 10.1001/jamanetworkopen.2023.22650 (PMC10336617; doi:10.1001/jamanetworkopen.2023.22650)
Supplement: Supplement 2. — Data Sharing Statement [file jamanetwopen-e2322650-s002.pdf]

Data Sharing Statement

McCabe. Cocaine or Methamphetamine Use During Young Adulthood Following Stimulant Use for Attention-Deficit/Hyperactivity Disorder During Adolescence. *JAMA Netw Open*. Published July 11, 2023. doi:10.1001/jamanetworkopen.2023.22650

Data
Data available: No

Additional Information
Explanation for why data not available: This study contains the results of secondary analysis of the USA Monitoring the Future (MTF) surveys. The authors followed university and MTF protocol regarding access to and analysis of the data for this study. Data is not available without written consent from MTF and interested researchers can apply for MTF panel data access through the US National Addiction & HIV Data Archive Program (NAHDAP) at the University of Michigan. More information may be found here: https://www.icpsr.umich.edu/web/NAHDAP/studies/37072
